# Full-body pose reconstruction and correction in virtual reality for rehabilitation training

**DOI:** 10.3389/fnins.2024.1388742

**Published:** 2024-04-04

**Authors:** Xiaokun Dai, Zhen Zhang, Shuting Zhao, Xueli Liu, Xinrong Chen

**Affiliations:** ^1^Academy for Engineering & Technology, Fudan Universiry, Shanghai, China; ^2^Shanghai Key Laboratory of Medical Image Computing and Computer Assisted Intervention, Shanghai, China; ^3^Baoshan Branch of Ren Ji Hospital, Shanghai Jiao Tong University School of Medicine, Shanghai, China; ^4^EYE & ENT Hospital of Fudan University, Shanghai, China

**Keywords:** rehabilitation training, virtual reality, full-body pose reconstruction, deep learning, Multilayer Perceptron (MLP)

## Abstract

Existing statistical data indicates that an increasing number of people now require rehabilitation to restore compromised physical mobility. During the rehabilitation process, physical therapists evaluate and guide the movements of patients, aiding them in a more effective recovery of rehabilitation and preventing secondary injuries. However, the immutability of mobility and the expensive price of rehabilitation training hinder some patients from timely access to rehabilitation. Utilizing virtual reality for rehabilitation training might offer a potential alleviation to these issues. However, prevalent pose reconstruction algorithms in rehabilitation primarily rely on images, limiting their applicability to virtual reality. Furthermore, existing pose evaluation and correction methods in the field of rehabilitation focus on providing clinical metrics for doctors, and failed to offer patients efficient movement guidance. In this paper, a virtual reality-based rehabilitation training method is proposed. The sparse motion signals from virtual reality devices, specifically head-mounted displays hand controllers, is used to reconstruct full body poses. Subsequently, the reconstructed poses and the standard poses are fed into a natural language processing model, which contrasts the difference between the two poses and provides effective pose correction guidance in the form of natural language. Quantitative and qualitative results indicate that the proposed method can accurately reconstruct full body poses from sparse motion signals in real-time. By referencing standard poses, the model generates professional motion correction guidance text. This approach facilitates virtual reality-based rehabilitation training, reducing the cost of rehabilitation training and enhancing the efficiency of self-rehabilitation training.

## 1 Introduction

Existing statistical data indicate that an increasing number of people are now experiencing mobility impairments due to accidents, illness, or aging, thereby demanding the need for rehabilitation (Postolache et al., 2020). Rehabilitation training encompasses a series of intervention exercises aimed at aiding in the recovery of compromised motor functions. A pivotal aspect of this process involves tailored movement exercises conducted by a doctor or physical therapist. Early and intensive rehabilitation training proves more efficacious in facilitating the recovery of patients' motor abilities (Postolache et al., 2020). However, the demand for patients to attend hospitals or rehabilitation centers for rehabilitation training presents additional challenges for those already grappling with mobility difficulties. Furthermore, the high cost associated with rehabilitation training becomes a financial impediment for certain patients. In this context, the emergence of virtual reality-based rehabilitation methods becomes apparent. These methods allow patients to engage in a more convenient and economical rehabilitation option through personalized virtual reality devices. By offering real-time user pose reconstruction and employing immersive interactive methods, virtual reality technology can provide patients with increased sensory stimulation and a more immersive environment during rehabilitation training (Adamovich et al., 2009). Existing research has shown that compared to conventional physical therapy, virtual reality-based rehabilitation training is more effective in promoting gait recovery in patients with Parkinson's disease (Feng et al., 2019).

However, common virtual reality devices can only accurately reconstruct the poses of user's head and hands through head-mounted displays and handheld controllers. This limitation is insufficient for full body rehabilitation training. Therefore, virtual reality-based rehabilitation methods often require additional wearable body measurement sensors to capture patients' movements (Huang et al., 2018; Jiang Y. et al., 2022), such as motion sensors for the legs and waist, gait detection devices, and more. Unfortunately, for patients, this not only represents an additional expense, but wearing extra sensors may also lead to physical discomfort. Moreover, patients' unprofessional handling of these sensors can result in tracking inaccuracies and affecting the effectiveness of the rehabilitation training. Hence, the studies that leverage the most prevalent virtual reality devices, using the sparse motion signals from the head and hands to reconstruct full body poses, demonstrate an effective solution for virtual reality-based rehabilitation training methods.

In addition, regardless of the form of rehabilitation, the quality assessment and precise guidance of patients' recovery movements are crucial (Qiu et al., 2022). This directly influences the effectiveness of patient's recovery. When patients participate in rehabilitation training at hospitals or rehabilitation facilities, doctors can assist by correcting their inaccurate movements, ensuring that their movements fall within the normal range to achieve the desired rehabilitation effects. This correction helps prevent secondary injuries resulting from incorrect movements. However, in virtual reality-based rehabilitation, there is currently no universally recognized solution to reasonably evaluate the quality of patients' rehabilitation movements (Qiu et al., 2022). Furthermore, there is no method to authentically simulate a doctor's supervision to aid patients in correcting rehabilitation movements. Therefore, proposing effective methods for correcting rehabilitation movements and providing appropriate movement guidance is crucial to advancing research in virtual reality-based rehabilitation.

**Figure 1:**
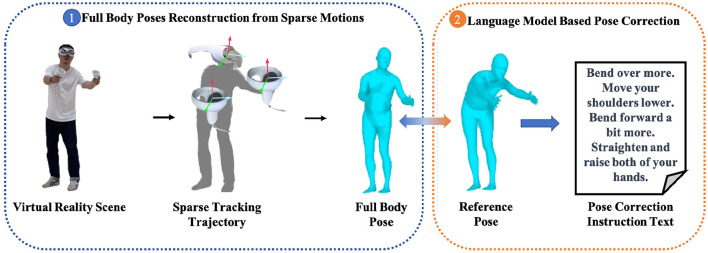
The pipeline of the proposed virtual reality-based rehabilitation training method.

To address the aforementioned issues, we propose a virtual reality-based rehabilitation method. As shown in Figure 1, this method utilizes commonly available virtual reality hardware devices to reconstruct full-body poses of patients. Then, a pose correction module based on a natural language model is employed to assess patients' movements, which generates specific movement correction guidance text by referencing standard movements. Specifically, to meet the requirement of accuracy, real-time performance and smoothness in full-body poses reconstruction, a deep learning-based model is introduced, comprising a multi-scale temporal feature switch module and a stacked MLP Blocks. The multi-scale temporal feature switch module expands the model's temporal receptive field, improving the accuracy and the smoothness of full-body poses reconstruction while ensuring real-time performance and model light-weighting. Subsequently, the reconstructed poses and the reference standard poses are input into the poss correction module, which assesses the reconstructed poses and outputs the movement correction guidance text by utilizing a natural language model. Comprehensive experimental results demonstrate that the proposed method can provide more accurate full-body poses reconstruction and more intelligent movement guidance for virtual reality-based rehabilitation training.

## 2 Related works

### 2.1 Full-body pose reconstruction from sparse motion signals

In recent years, the reconstruction of full-body poses using sparse motion signals from virtual reality devices, specifically head-mounted displays and handheld controllers, has become a focal point in research within the realms of virtual reality and the metaverse. Ahuja et al. introduced a convolutional neural network to extract features from sparse motion signals and utilized a K-nearest neighbors (KNN)—based method, employing interpolation algorithms to reconstruct the full-body poses from a limited motion database (Ahuja et al., 2021). However, this method heavily relies on the motion database, exhibiting poor generalization capabilities. In subsequent studies, novel deep learning models such as variational autoencoders (Pavlakos et al., 2019), long short-term memory networks (Yu et al., 2019), and transformers (Jiang J. et al., 2022; Luo et al., 2022; Zhang X. et al., 2023) have been applied to extract motion features from sparse motion signals, significantly enhancing the accuracy of full-body poses reconstruction. In recent studies, based on Multilayer Perceptron (MLP), a diffusion model has been employed to further optimize the reconstructed motion sequences, effectively alleviating the phenomenon of joint jitter (Du et al., 2023). However, the adoption of the diffusion model has substantially increased the computational demands and inference time of the model.

### 2.2 Pose evaluation for healthcare application

With the development of electronic information and computing technology, studies focusing on health applications, particularly the evaluation of human body poses during rehabilitation training, has been recently explored. Martınez et al. utilized depth cameras to capture the ground-truth human rehabilitation postures and quantitatively evaluated the accuracy of commonly used pose reconstruction algorithms (Martınez, 2019) in reconstructing rehabilitation postures (Hernández et al., 2021). Kidziński et al. (2020) introduced a neural network to quantitatively evaluate clinically relevant motion parameters from patients' motion videos. Xu et al. (2022) employed multi-view videos for the evaluation of musculoskeletal patients' motion poses. Liao et al. (2020) combining the Long Short-Term Memory (LSTM), feature pyramids, and other deep learning methods, which designed the first rehabilitation posture quality evaluation method based on deep learning. Tang (2020) introduced a segmentation module to the posture evaluation network, significantly enhancing the accuracy of scores in evaluating the quality of rehabilitation postures. Bruce et al. employed graph convolutional networks to assess the severity of Alzheimer's disease in patients through motion videos (Bruce et al., 2021). However, the quantitative evaluation metrics of these methods are exclusive to proficient medical professionals for clinical evaluations, limiting their applicability for patients to comprehend the status of their rehabilitation training and make corrections.

To address the aforementioned issues, Qiu et al. (2022) devised a pose matching network, which achieves alignment and correction of poses between the trainers' pose and the standard poses, providing trainers with visualized movement guidance through Class Activation Maps (CAM). Despite having a certain foundation in research, the majority of these methods heavily rely on computer vision and are impractical for virtual reality-based rehabilitation. Moreover, there remains a dearth of intuitive and effective guidance for patients in evaluating their poses, such as the guidance provided by medical professional.

### 2.3 3D human poses and natural language models

In recent years, Transformer-based natural language processing models have achieved remarkable success in various fields. The following will introduce datasets that combine human poses with natural language processing models and showcase astonishing applications. The AMASS dataset (Mahmood et al., 2019) has collected motion data for numerous 3D human poses in the form of SMPL (Loper et al., 2023). Then, BABEL (Punnakkal et al., 2021) and HumanML3D (Guo et al., 2022), building on the AMASS dataset, provide free-from textual descriptions for its sequence data. These datasets focus more on describing the entire action sequences rather than the semantic information of each single-frame pose. Consequently, they are more suitable for tasks for generating action sequences (Zhang J. et al., 2023) or describing motions from videos. To address the gap in independent human pose semantic descriptions, PoseScript (Delmas et al., 2022) provides descriptions for each single-frame human poses from some subsets of the AMASS dataset. In further research, FixMyPose (Kim et al., 2021) and PoseFix (Delmas et al., 2023) can connect two different poses and generate textural information for pose correction. Unlike FixMyPose, which generates textual annotations from rendering 2D images, the PoseFix directly generates text explanations based on the 3D human pose data. This proves to be a more suitable and more potent solution for the virtual reality-based rehabilitation training.

## 3 Methods

### 3.1 Overview

Reconstructing full-body movements from sparse motion inputs is quite challenging. Sparse motion signals from the upper body cannot effectively constrain the movements of the lower body. As a result, the reconstruction of lower body poses may inevitably exhibit anomalies such as joint jitter and floor penetration, significantly affecting the user's experience in virtual reality. In previous studies (Du et al., 2023), one-dimensional convolution with temporal awareness-based diffusion models was employed to reduce joint jitter, noticeably enhancing the quality and fluency of full-body posture reconstruction. However, the diffusion model requires multiple inference steps, leading to longer model inference times that do not meet the real-time requirements of virtual reality applications. Moreover, when using only its MLP backbone network, joint jitter phenomena remain unresolved.

**Figure 2:**
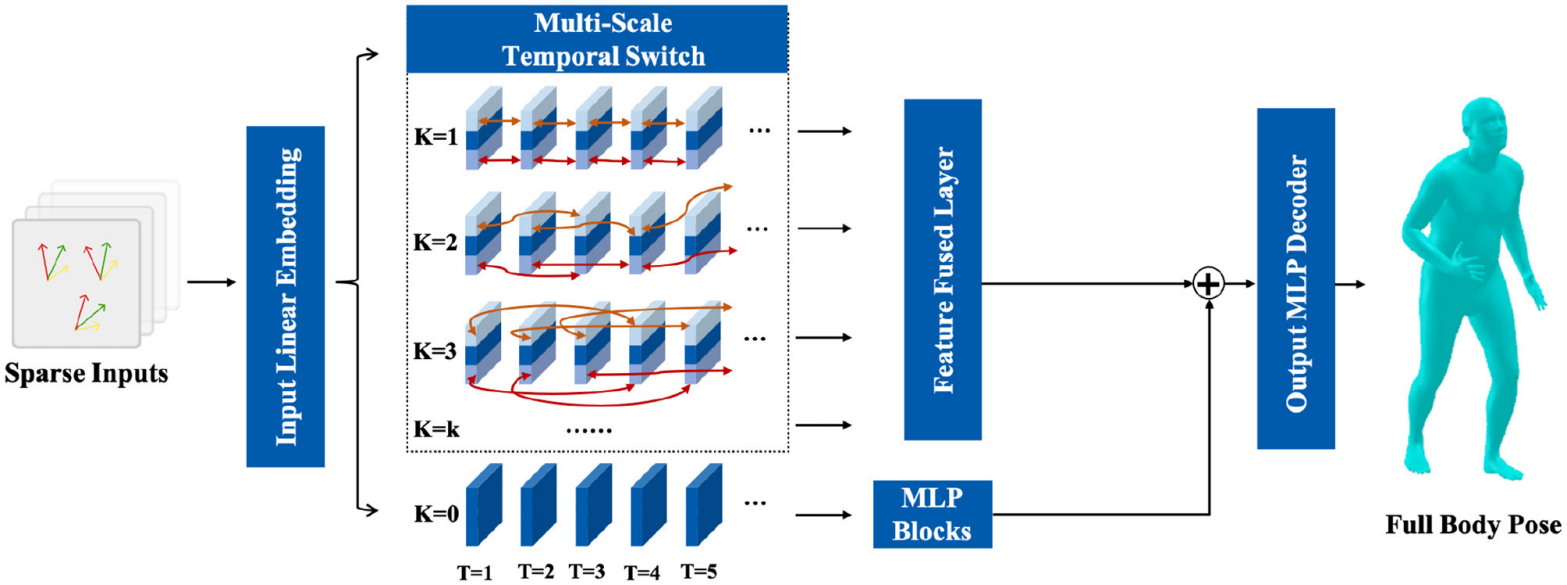
The detailed structure of proposed full-body pose reconstruct module and temporal switch module.

Therefore, as shown in Figure 2, a full-body pose reconstruction network based on a multi-scale temporal switch module is proposed. The sparse motion signals are input to an MLP layer for feature embedding and then input into the multi-scale temporal switch module for aggregation of features across different time scales. Subsequently, the original features are fed into a stacked MLP module and the features aggregated at different time scales are fed into a MLP layer. Finally, the depth features from different scales are aggregated, fused with the original features, and input into an MLP layer for the reconstruction of full-body poses.

### 3.2 Full-body pose reconstruct module

#### 3.2.1 Data preparation

To reconstruct the full body's poses, sparse motion signals are acquired from the Inertial Measurement Unit (IMU) devices on the virtual reality headset and handheld controllers. Each signal at every position includes global positional information *p*^1×3^ and rotational information θ^1×3^ about the three axes. For a more refined reconstruction outcome, predicting the human body's motion posture at time *t* poses from a certain time interval *T* before time *t* is aggregated and jointly fed into the network. Therefore, the full-body joint pose $U_{\text{full}}^{\text{joints}}$ is obtained by applying the mapping function Φ to the set of sparse inputs {_*p*_*i*_, θ_*i*_}1:*T*_, as shown in Equation 1:


(1) $$\begin{array}{l} U_{\text{full}}^{\text{joints}}=\Phi \left(\overset{n}{\underset{i=1}{⋃}}{\left\{p_{i},\theta_{i}\right\}}_{1:T}\right) \end{array}$$

where *n* represents the number of sparse inputs, *h* is the quantity of full-body joints, and *T* is the count of continuous motion frames observed from the past.

To enable the model to comprehensively learn features from sparse motion signals, the following preprocessing steps are employed. The backward finite difference method is employed to initiate the calculation of linear velocity *v*^1×3^, as shown in Equation 2:


(2) $$\begin{array}{l} v_{t}=p_{t}-p_{t-1} \end{array}$$

Subsequently, the angular velocity $\Omega_{t}^{1\times 6}$ is defined by considering the orientation matrices **R** of the sparse input (Jiang J. et al., 2022), as shown in Equation 3:


(3) $$\begin{array}{l} \Omega_{t}={\text{R}}_{t-1}^{-1}{\text{R}}_{t} \end{array}$$

These matrices are initially derived from the θ^1×3^ representation, which are converted to the rotation matrix **R**^3×3^ using the conversion as previous studies (Zhou et al., 2019; Jiang J. et al., 2022). Following this, the last row of **R** is disregarded to yield the 6D rotation representation $w_{t}^{1\times 6}$.

Consequently, each input at time frame *t*_*i*_ comprises four vectors: *p*_*i*_, *v*_*i*_, Ω_*i*_, and *w*_*i*_. This input feature is structured as Equation 4:


(4) $$\begin{array}{l} x_{t}=\left[p_{t}^{1},v_{t}^{1},w_{t}^{1},\Omega_{t}^{1},p_{t}^{2},v_{t}^{2},w_{t}^{2},\Omega_{t}^{2},p_{t}^{3},v_{t}^{3},w_{t}^{3},\Omega_{t}^{3}\right] \end{array}$$

As a result, all independent signals *x*_*t*_ within the time interval *T* are concatenated along the temporal dimension to form the input signal **X**, as shown in Equation 5:


(5) $$\begin{array}{l} \text{X}=\left[x_{1},x_{2},x_{3},\ldots ,x_{T}\right],\text{X}\in {\mathbb{R}}^{B\times T\times F} \end{array}$$

where *B* represent the batch size, *T* signifies the length of the temporal sequences, and *F* denotes the feature dimension. Therefore, the feature dimension *F* of the input tensor **X** amounts to 54.

#### 3.2.2 Multi-scale temporal switch module

In previous research (Du et al., 2023), networks equipped with one-dimensional temporal convolutions are employed to enhance the model's temporal awareness, aiming for improving reconstruction of full-body poses. Additionally, the powerful generative ability of diffusion model is utilized to further optimize the reconstructed pose sequences, significantly reducing the occurrence of joint jitter. However, despite the application of Denoising Diffusion Implicit Model (DDIM) technology (Ho et al., 2020), the diffusion model still necessitates five repeated inference steps to obtain the final predictions, which fails to meet the real-time requirements of virtual reality-based rehabilitation training.

To address these issues, a multi-scale temporal switch module based on two-dimensional time sequences is devised. This module comprises multiple branches at different temporal scales, aiding the model in capturing subtle temporal features within the sparse motion signals.

Initially, the preprocessed sparse motion signals **X** are fed into a Linear Layer for preliminary feature embedding, as shown in Equation 6:


(6) $$\begin{array}{l} F=LinearEmbedding\left(X\right) \end{array}$$

where the *LinearEmbedding* is a linear layer with an input dimension of 54 and an output dimension of 256. As shown in Figure 2, the module comprises *K* branches representing different temporal switch scale. For each time slice *T* = *t* alone the temporal dimension, the feature **F**^*t*^ is partitioned into three segments along the feature dimensions, such as $f_{1}^{t}$, $f_{2}^{t}$, and $f_{3}^{t}$. Here, as shown in Equation 7, the $f_{1}^{t}$ and $f_{3}^{t}$ are the first *N* features and the last *N* features alone the feature dimension, respectively:


(7) $$\begin{array}{l} F^{t}=\left(f_{1}^{t},f_{2}^{t},f_{3}^{t}\right) \end{array}$$

where **F**^*t*^ ∈ ℝ^*B*×1×*F*^, $f_{1}^{t}\in {\mathbb{R}}^{B\times 1\times F/8}$, $f_{2}^{t}\in {\mathbb{R}}^{B\times 1\times 3F/4}$, and $f_{3}^{t}\in {\mathbb{R}}^{B\times 1\times F/8}$.

Inspired by previous study (Zheng et al., 2022), we conduct **K** forward feature exchange modules alone the temporal direction for each feature slice **F**^*t*^. In the branch where **K** = *k*, we exchange the features $f_{1}^{t}$ and $f_{3}^{t}$ in the feature slice **F**^*t*^ with the corresponding features $f_{1}^{t+k}$ and $f_{3}^{t+k}$ in the feature slice **F**^*t*+*k*^, where **F**^*t*+*k*^ represents the feature slice at a temporal distance of *k* frames, as shown in Equation 8:


(8) $$\begin{array}{l} F_{k}^{t}=\left(f_{1}^{t+k},f_{2}^{t},f_{3}^{t+k}\right) \end{array}$$

After the exchange of features slice for all time frames *T* = *t*, we concatenate all the time slices along the temporal dimension to obtain the output **F**_*k*_ of the feature exchange module **K** = *k* in Equation 9:


(9) $$\begin{array}{l} F_{k}=\left\{F_{k}^{1},F_{k}^{2},\ldots ,F_{k}^{t}\right\} \end{array}$$

Finally, features from different branches are input into an MLP layer for feature fusion, as shown in Equation 10:


(10) $$\begin{array}{l} F_{switched}=OutPutLinear\left\{F_{1}⊙F_{2}⊙\ldots ⊙F_{k}\right\} \end{array}$$

where ⊙ represents the concatenate operation and ${\text{F}}_{switched}\in {\mathbb{R}}^{B}\times T\times F$, and the *OutPutLinear* is a linear layer with an input dimension of K*F and an output dimension of *F* and the *SiLu* activative function.

The difference between previous study and ours is that our approach solely employs forward switch along the temporal direction, refraining from bidirectional switch. Our rationale lies in the fact that bidirectional switch necessitates a greater number of feature switch operations for a limited enhancement. Given the constrained computational capacity of the virtual reality devices and the stringent demands for real-time processing, we opt for unidirectional propagation.

#### 3.2.3 MLP based blocks

**Figure 3:**
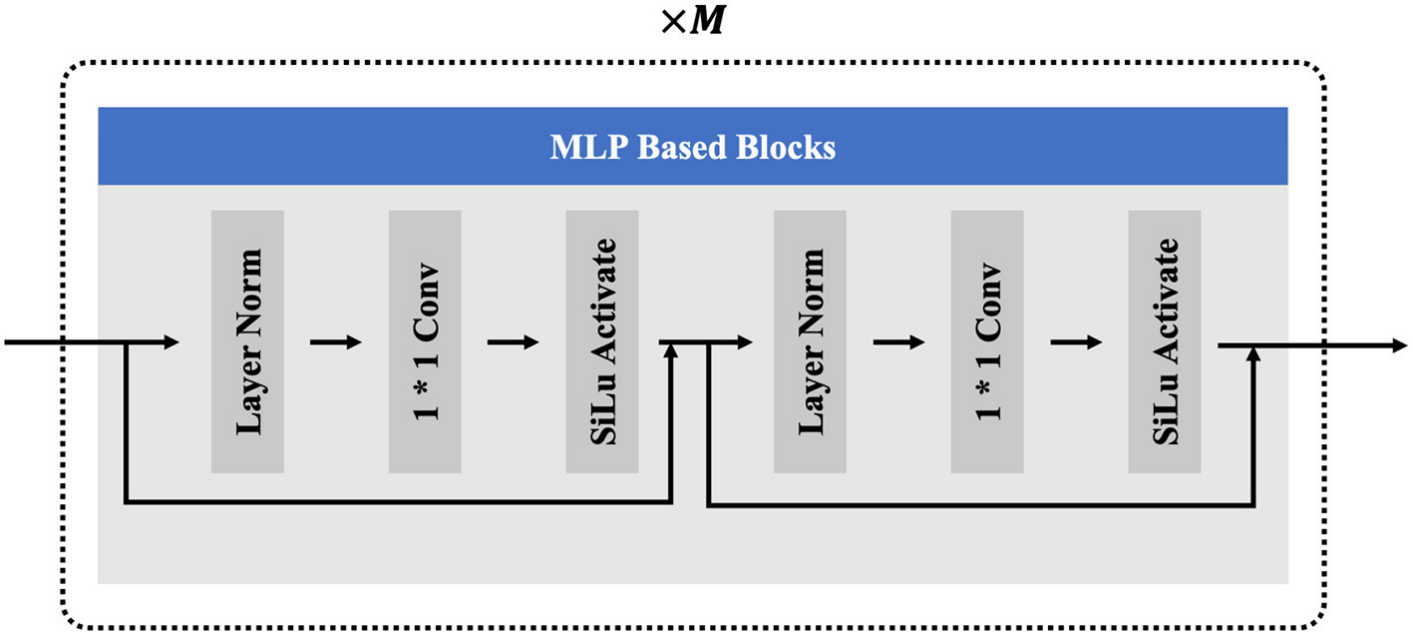
The components of the MLP based blocks.

In the recent research (Du et al., 2023; Guo et al., 2023), the potential of MLP-based networks in full-body poses reconstruction tasks has been demonstrated. The MLP-based networks can effectively learn complex non-linear mapping relationships of input features, facilitating efficient feature learning and data representation (Guo et al., 2023). Additionally, the MLP networks possess the advantage of lightweight design, meeting the real-time requirements of our tasks. Considering the demands for both real-time processing and accuracy of the reconstructed poses, our model only employs several commonly used and effective modules in the field of deep learning, including fully connected layers, Silu activation function, one-dimensional convolution alone the temporal dimension with a size of 1, and the layer normalization. Specifically, the one-dimensional convolution layer is utilized to aggregate the temporal features from the entire pose sequence, while the other modules operate on the feature dimension to help the network alleviate gradient vanishing and overfitting phenomena. The structure of the MLP-based blocks is shown in Figure 3. To better extract features from sparse motion signals, the MLP-based blocks are stacked in *M* layers as in the study by Du et al. (2023).

As the proposed temporal switch module affects the spatial information of the original motion signals, the original feature *f* is preserved and fed into the aforementioned MLP-based Blocks for feature extraction in Equation 11:


(11) $$\begin{array}{l} F_{0}=MLPBlocks\left(f\right) \end{array}$$

where ${\text{F}}_{0}\in {\mathbb{R}}^{B}\times T\times F$.

Finally, the output feature **F**_0_ and the temporal switched feature **F**_*switched*_ are aggregated and input into the output MLP Layer to reconstruct the poses of 22 joints (excluding the joints of the palms) in the SMLP human pose model, achieving the reconstruction from sparse motion signals to full body poses, as shown in Equation 12:


(12) $$\begin{array}{l} F_{output}=Linear\left(F_{0}⊕F_{switched}\right) \end{array}$$

where the ⊕ represents tensor addition operation and ${\text{F}}_{output}\in {\mathbb{R}}^{B}\times T\times 132$.

### 3.3 NLP-based pose correction module

**Figure 4:**
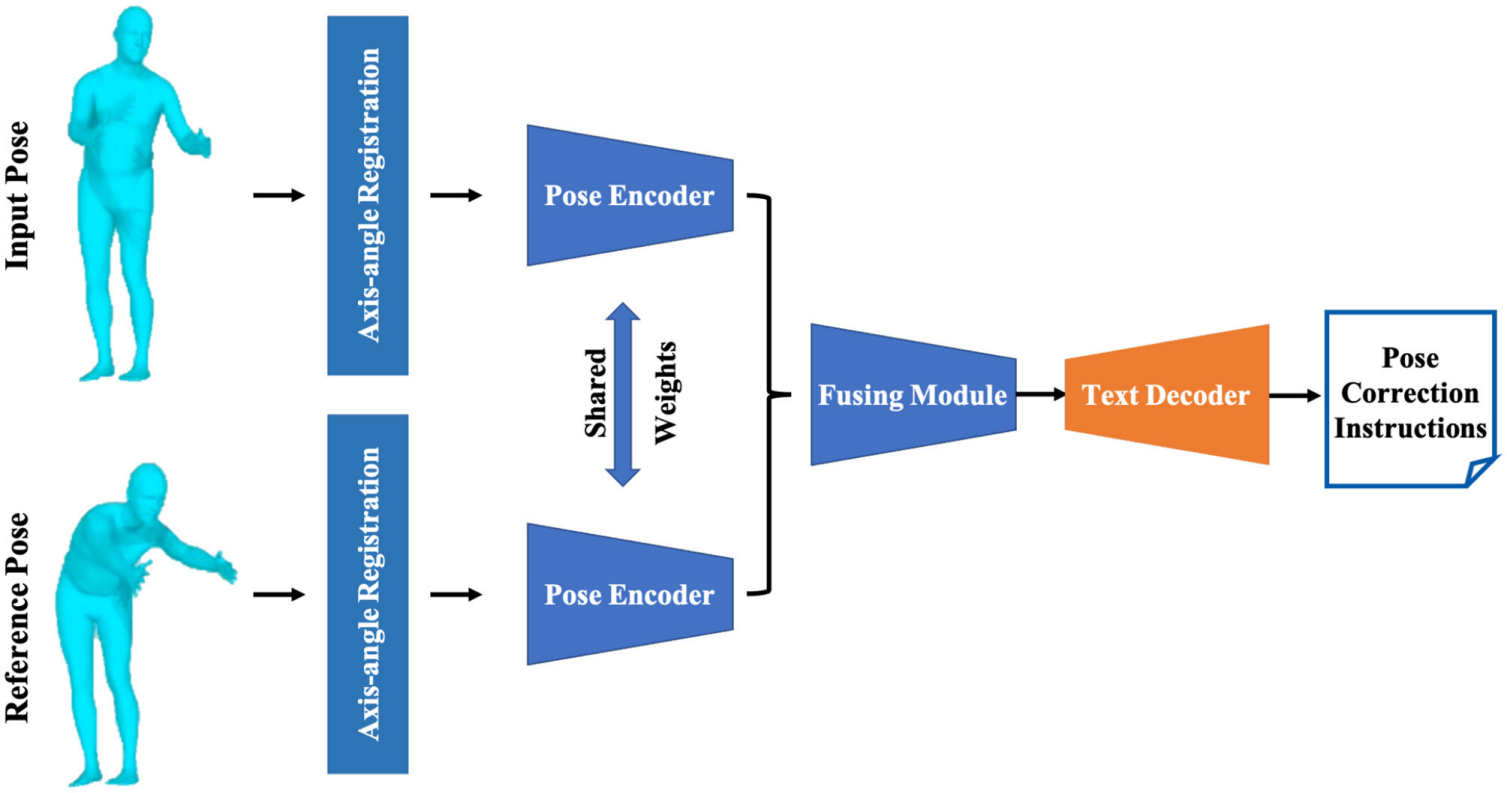
The structure of the pose correction module, which outputs pose correction guidance text for the users by contrasting the reconstructed pose with the standard reference pose.

In this section, the state-of-the-art pose evaluation method, PoseFix (Delmas et al., 2023), is employed to compare the reconstructed full body poses and the standard poses, and generate professional motion correction guidance text. We will briefly elucidate how the reconstructed pose *Pose*_*A*_ of the patient is matched to the target pose *Pose*_*B*_ and modeled as correction guidance text. As shown in Figure 4, the rotation angles of the root joint of *Pose*_*A*_ are aligned with the corresponding rotation angles of *Pose*_*B*_. Subsequently, a Transformer-based auto encoder (Kingma and Welling, 2013) is utilized to extract independent 32-dimensional embedded features from *Pose*_*A*_ and *Pose*_*B*_. It is noteworthy that the *Pose*_*A*_ and *Pose*_*B*_ share the weights of the auto encoder. Next, the TIRG network (Vo et al., 2019), a widely applied module for compositional learning, is used to merge latent features from the embedded features of *Pose*_*A*_ and *Pose*_*B*_. The TIRG (Vo et al., 2019) network comprises a gate network consisting of two MLP layers and two learnable weights, which is designed to retain the primary motion features and introduce additional improvement through residual connections. As shown in Equation 13:


(13) $$\begin{array}{l} F_{prompt}=w_{f}FC_{f}\left(\left[\text{a},\text{m}\right]\right)⊙\text{a}+w_{g}FC_{g}\left(\left[\text{a},\text{m}\right]\right) \end{array}$$

where *FC*_*f*_ and *FC*_*g*_ are MLP layers, and their weights are balanced by learnable parameters *w*_*f*_ and *w*_*g*_.

Finally, the fused features **F**_*prompt*_ are fed into a Transformer-based auto-regressive model, serving as a prompt to guide the natural language processing model in generating motion correction guidance text. In the decoding process of the Transformer-based auto-regressive model, the input feature **F**_*prompt*_ is concatenated with a vector *F*_*caption*_, composed entirely of ones, serving as additional positional encoding. This combined input is then fed into the Transformer model. Leveraging the attention mechanism of the Transformer, the prompt is decoded into a probability distribution of text embeddings, and the first text result *T*_1_ is obtained through the softmax function. Subsequently, *T*_1_ is integrated into *F*_*caption*_, concatenated again with the input feature **F**_*prompt*_, and fed into the Transformer model to obtain the second text result *T*_2_ with the highest probability. This iterative process continues, employing the method of iterative greedy decoding, until the entire sequence is decoded.

## 4 Experiments

### 4.1 Training details

To train the full-body pose reconstruction model based on sparse motion signals, three subsets of the AMASS dataset—CMU (Carnegie Mellon University), MPI-HDM05 (Max Planck Institute Human Motion Database 2005; Müller et al., 2007), and BioMotionLab-NTroje (Troje, 2002) are employed for model training and test. Specifically, we obtain 2,074, 215, and 3,061 motion sequences from these three subsets, covering commonly used actions in virtual reality such as walking, running, jumping, dancing, kicking, tool manipulation, and social behaviors and interpersonal interactions. Out of 5,350 motion sequences, 536 are randomly selected for model validation, with the remaining 4,814 used for model training. These motion sequences are stored in the format of SMPL model parameters, encompassing 156-dimensional joint motion parameters.

To emulate the hardware configuration of virtual reality devices, we extract the motion parameters of the head joint and wrists of both hands, inputting them into the model, and reconstruct the motion parameters of 22 body joints (excluding the joints of the palms). To ensure a fair comparison with previous methods, consistent experimental parameters are employed: the stacking layers of the MLP module *M* are set to 12, and the feature dimension *F* was set to 512. Both training and testing were conducted on an NVIDIA 4090 GPU using the PyTorch framework (Paszke et al., 2019).

For the natural language processing model-based pose correction module, we make no modifications and training to the PoseFix model. In PoseFix, a pipeline based on PoseScript is employed to compare the distance variations between multiple 3D keypoints for 135 k pairs of different actions. The resulting data are organized in structural order, forming the 135 k action correction guidance text dataset. This dataset is utilized for training the pose correction model. Additionally, the frozen DistillBERT (Sanh et al., 2019) is employed for word embedding. Instead, we directly utilize publicly available model weights, as experimental results have already demonstrated that this method accurately evaluates differences between two poses and generates precise correction guidance text.

### 4.2 Evaluation metrics

To validate the effectiveness of the proposed method, the following evaluation metrics are employed to assess the model's performance and compare it with previous state-of-the-art methods (Du et al., 2023): Mean Per Joint Rotation Error (degrees; MPJRE) and Mean Per Joint Position Error (cm; MPJPE) measures the average relative rotation error and position error for each joints, which indicated the absolute errors of the model predictions. While the Mean Per Joint Velocity Error (cm/s; MPJVE) measures the average velocity error for the joints' positions, the Jitter (Yi et al., 2022) evaluates the mean jerk (change in acceleration over time; Du et al., 2023) of the joints in global space. These metrics can measure the smoothness of reconstructed poses, which directly relates to the user's overall experience. Specifically, jitter delineates the rate of change of acceleration in joint positions, serving as an indicator of the degree to which abrupt changes occur in joint positioning. Consequently, it proves valuable in characterizing and analyzing the dynamic aspects of motion, facilitating an evaluation metric of the smoothness of reconstructed poses (Flash and Hogan, 1985). The jitter is calculated as Equation 14:


(14) $$\begin{array}{l} Jitter=\frac{d^{2}p}{dt^{2}} \end{array}$$

where *p* represents the joint position and *t* denotes the time. By computing the second derivative of each joint position with respect to time, jitter can be derived.

### 4.3 Evaluation results

In Table 1, we present the quantitative comparison results between our proposed model and the state-of-the-art method AGRoL (Du et al., 2023). As shown in Table 1, our approach demonstrates improvements across various metrics compared with the AGRoL's MLP-based backbone method. Moreover, the phenomenon of joint jitter has been noticeably mitigated. In comparison to AGRoL's diffusion model method, we maintain a comparable prediction accuracy, albeit with less pronounced joint jitter. However, our method requires only 60.4% of the model's parameters compared with this method, enhancing its practical applicability.

**Table 1:** Comparison of our approach with state-of-the-art methods on the subsets of AMASS.

**Method**	**MPJPE**	**MPJRE**	**MPJVE**	**Jitter**	**Parameters (M)**
AGRoL-MLP	3.93	2.69	22.85	13.01	3.73
AGRoL-Diffusion	3.71	2.66	18.59	7.26	7.48
Ours	3.79	2.69	20.94	11.53	4.52

**Figure 5:**
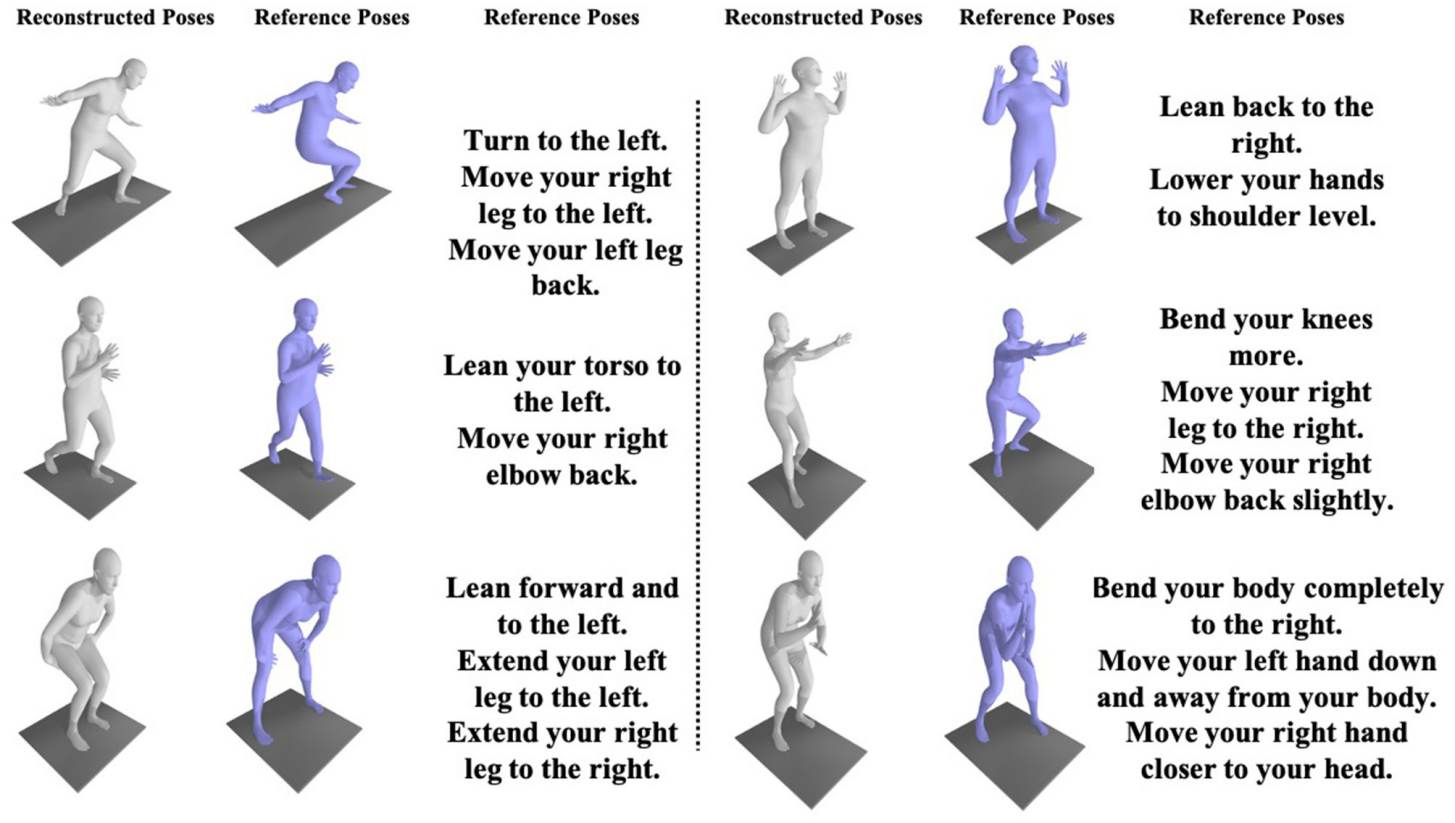
Partial results of the virtual reality-based rehabilitation training method indicate that users can correct their poses with guidance from the pose correction text.

In Figure 5, we showcase comprehensive applications of virtual reality-based rehabilitation and partial action guidance. As shown in the figure, the reconstructed poses and target poses can be accurately evaluated by the PoseFix network, yielding intuitive, detailed, and precise action guidance.

## 5 Conclusion

Current rehabilitation training requires patients, who already face mobility challenges, to visit rehabilitation centers for treatment by physical therapists. This proves to be difficult and costly for patients. To enable patients to undergo precise, efficient, and cost-effective rehabilitation training in the comfort of their homes using their virtual reality devices, this study introduces a novel approach that utilizes sparse motion signals from VR devices, specifically head-mounted displays and hand controllers, to reconstruct full-body poses. Unlike existing methods that focus on clinical metrics for doctors, our method employs a natural language processing model to contrast reconstructed poses with standard poses. This process provides efficient pose correction guidance in the form of natural language, offering a more accessible and personalized approach to movement guidance for patients.

The quantitative and qualitative results demonstrate the effectiveness of the proposed method in real-time reconstruction of accurate full-body poses. By referencing standard poses, the model generates professional motion correction guidance text, facilitating virtual reality-based rehabilitation training. This approach not only reduces the cost of rehabilitation training but also enhances the efficiency of self-rehabilitation training, addressing the challenges faced by patients seeking timely and accessible rehabilitation.

## Data availability statement

The original contributions presented in the study are included in the article/supplementary material, further inquiries can be directed to the corresponding authors.

## Author contributions

XD: Conceptualization, Investigation, Methodology, Writing – original draft. ZZ: Investigation, Writing – original draft. SZ: Software, Visualization, Writing – original draft. XL: Supervision, Writing – review & editing. XC: Supervision, Writing – review & editing.

## References

[B1] Adamovich S. V. Fluet G. G. Tunik E. Merians A. S. (2009). Sensorimotor training in virtual reality: a review. NeuroRehabilitation 25, 29–44. 10.3233/NRE-2009-0497 19713617 PMC2819065

[B2] Ahuja K. Ofek E. Gonzalez-Franco M. Holz C. Wilson A. D. (2021). Coolmoves: user motion accentuation in virtual reality. Proc. ACM Interact. Mob. Wear. Ubiquit. Technol. 5, 1–23. 10.1145/3463499

[B3] Bruce X. Liu Y. Chan K. C. Yang Q. Wang X. (2021). Skeleton-based human action evaluation using graph convolutional network for monitoring Alzheimer's progression. Pat. Recogn. 119:108095. 10.1016/j.patcog.2021.108095

[B4] Delmas G. Weinzaepfel P. Lucas T. Moreno-Noguer F. Rogez G. (2022). Posescript: 3D human poses from natural language, in European Conference on Computer Vision (Berlin: Springer), 346–362.

[B5] Delmas G. Weinzaepfel P. Moreno-Noguer F. Rogez G. (2023). Posefix: correcting 3D human poses with natural language, in Proceedings of the IEEE/CVF International Conference on Computer Vision, 15018–15028.

[B6] Du Y. Kips R. Pumarola A. Starke S. Thabet A. Sanakoyeu A. (2023). Avatars grow legs: generating smooth human motion from sparse tracking inputs with diffusion model, in Proceedings of the IEEE/CVF Conference on Computer Vision and Pattern Recognition, 481–490.

[B7] Feng H. Li C. Liu J. Wang L. Ma J. Li G.. (2019). Virtual reality rehabilitation versus conventional physical therapy for improving balance and gait in Parkinson's disease patients: a randomized controlled trial. Med. Sci. Monit. 25:4186. 10.12659/MSM.916455 31165721 PMC6563647

[B8] Flash T. Hogan N. (1985). The coordination of arm movements: an experimentally confirmed mathematical model. J. Neurosci. 5, 1688–1703. 4020415 10.1523/JNEUROSCI.05-07-01688.1985 PMC6565116

[B9] Guo C. Zou S. Zuo X. Wang S. Ji W. Li X.. (2022). Generating diverse and natural 3D human motions from text, in Proceedings of the IEEE/CVF Conference on Computer Vision and Pattern Recognition, 5152–5161.

[B10] Guo W. Du Y. Shen X. Lepetit V. Alameda-Pineda X. Moreno-Noguer F. (2023). Back to MLP: a simple baseline for human motion prediction, in Proceedings of the IEEE/CVF Winter Conference on Applications of Computer Vision, 4809–4819.

[B11] Hernández Ó. G. Morell V. Ramon J. L. Jara C. A. (2021). Human pose detection for robotic-assisted and rehabilitation environments. Appl. Sci. 11:4183. 10.3390/app11094183

[B12] Ho J. Jain A. Abbeel P. (2020). Denoising diffusion probabilistic models. Adv. Neural Inform. Process. Syst. 33, 6840–6851.

[B13] Huang Y. Kaufmann M. Aksan E. Black M. J. Hilliges O. Pons-Moll G. (2018). Deep inertial poser: learning to reconstruct human pose from sparse inertial measurements in real time. ACM Trans. Graph. 37, 1–15. 10.1145/3272127.3275108

[B14] Jiang J. Streli P. Qiu H. Fender A. Laich L. Snape P.. (2022). Avatarposer: articulated full-body pose tracking from sparse motion sensing, in European Conference on Computer Vision (Berlin: Springer), 443–460.

[B15] Jiang Y. Ye Y. Gopinath D. Won J. Winkler A. W. Liu C. K. (2022). Transformer inertial poser: attention-based real-time human motion reconstruction from sparse IMUs. arXiv e-prints, arXiv–2203. 10.48550/arXiv.2203.15720

[B16] Kidziński Ł. Yang B. Hicks J. L. Rajagopal A. Delp S. L. Schwartz M. H. (2020). Deep neural networks enable quantitative movement analysis using single-camera videos. Nat. Commun. 11:4054. 10.1038/s41467-020-17807-z 32792511 PMC7426855

[B17] Kim H. Zala A. Burri G. Bansal M. (2021). Fixmypose: pose correctional captioning and retrieval, in Proceedings of the AAAI Conference on Artificial Intelligence, Vol. 35, 13161–13170.

[B18] Kingma D. P. Welling M. (2013). Auto-encoding variational bayes. arXiv preprint arXiv:1312.6114. 10.48550/arXiv.1312.6114

[B19] Liao Y. Vakanski A. Xian M. (2020). A deep learning framework for assessing physical rehabilitation exercises. IEEE Trans. Neural Syst. Rehabil. Eng. 28, 468–477. 10.1109/TNSRE.2020.2966249 31940544 PMC7032994

[B20] Loper M. Mahmood N. Romero J. Pons-Moll G. Black M. J. (2023). SMPL: a skinned multi-person linear model, in Seminal Graphics Papers: Pushing the Boundaries, Vol. 2, 851–866.

[B21] Luo J. Yuan M. Fu K. Wang M. Zhang C. (2022). Deep graph matching based dense correspondence learning between non-rigid point clouds. IEEE Robot. Automat. Lett. 7, 5842–5849. 10.1109/LRA.2022.3160237

[B22] Mahmood N. Ghorbani N. Troje N. F. Pons-Moll G. Black M. J. (2019). AMASS: archive of motion capture as surface shapes, in Proceedings of the IEEE/CVF International Conference on Computer Vision, 5442–5451.

[B23] Martınez G. H. (2019). Openpose: Whole-Body Pose Estimation. (Ph.D. thesis). Carnegie Mellon University, Pittsburgh, PA, United States.

[B24] Müller M. Röder T. Clausen M. Eberhardt B. Krüger B. Weber A. (2007). Mocap database HDM05. (Bonn: Institut für Informatik II, Universität Bonn), 2.

[B25] Paszke A. Gross S. Massa F. Lerer A. Bradbury J. Chanan G.. (2019). PyTorch: an imperative style, high-performance deep learning library. Adv. Neural Inform. Process. Syst. 32:12. 10.48550/arXiv.1912.01703

[B26] Pavlakos G. Choutas V. Ghorbani N. Bolkart T. Osman A. A. Tzionas D.. (2019). Expressive body capture: 3D hands, face, and body from a single image, in Proceedings of the IEEE/CVF Conference on Computer Vision and Pattern Recognition, 10975–10985.

[B27] Postolache O. Hemanth D. J. Alexandre R. Gupta D. Geman O. Khanna A. (2020). Remote monitoring of physical rehabilitation of stroke patients using IoT and virtual reality. IEEE J. Select. Areas Commun. 39, 562–573. 10.1109/JSAC.2020.3020600

[B28] Punnakkal A. R. Chandrasekaran A. Athanasiou N. Quiros-Ramirez A. Black M. J. (2021). Babel: bodies, action and behavior with english labels, in Proceedings of the IEEE/CVF Conference on Computer Vision and Pattern Recognition, 722–731.

[B29] Qiu Y. Wang J. Jin Z. Chen H. Zhang M. Guo L. (2022). Pose-guided matching based on deep learning for assessing quality of action on rehabilitation training. Biomed. Sign. Process. Contr. 72:103323. 10.1016/j.bspc.2021.103323

[B30] Sanh V. Debut L. Chaumond J. Wolf T. (2019). Distilbert, a distilled version of bert: smaller, faster, cheaper and lighter. arXiv preprint arXiv:1910.01108. 10.48550/arXiv.1910.01108

[B31] Tang D. (2020). Hybridized hierarchical deep convolutional neural network for sports rehabilitation exercises. IEEE Access 8, 118969–118977. 10.1109/ACCESS.2020.3005189

[B32] Troje N. F. (2002). Decomposing biological motion: a framework for analysis and synthesis of human gait patterns. J. Vis. 2:2. 10.1167/2.5.2 12678652

[B33] Vo N. Jiang L. Sun C. Murphy K. Li L.-J. Fei-Fei L.. (2019). Composing text and image for image retrieval-an empirical Odyssey, in Proceedings of the IEEE/CVF Conference on Computer Vision and Pattern Recognition, 6439–6448.

[B34] Xu W. Xiang D. Wang G. Liao R. Shao M. Li K. (2022). Multiview video-based 3-D pose estimation of patients in computer-assisted rehabilitation environment (CAREN). IEEE Trans. Hum. Machine Syst. 52, 196–206. 10.1109/THMS.2022.3142108

[B35] Yi X. Zhou Y. Habermann M. Shimada S. Golyanik V. Theobalt C.. (2022). Physical inertial poser (PIP): physics-aware real-time human motion tracking from sparse inertial sensors, in Proceedings of the IEEE/CVF Conference on Computer Vision and Pattern Recognition, 13167–13178.

[B36] Yu Y. Si X. Hu C. Zhang J. (2019). A review of recurrent neural networks: LSTM cells and network architectures. Neural Comput. 31, 1235–1270. 10.1162/neco_a_01199 31113301

[B37] Zhang J. Zhang Y. Cun X. Zhang Y. Zhao H. Lu H.. (2023). Generating human motion from textual descriptions with discrete representations, in Proceedings of the IEEE/CVF Conference on Computer Vision and Pattern Recognition, 14730–14740.

[B38] Zhang X. Chen X. Dai X. Di X. (2023). Dual attention poser: dual path body tracking based on attention, in Proceedings of the IEEE/CVF Conference on Computer Vision and Pattern Recognition, 2794–2803.

[B39] Zheng J. Liu X. Gu X. Sun Y. Gan C. Zhang J.. (2022). Gait recognition in the wild with multi-hop temporal switch, in Proceedings of the 30th ACM International Conference on Multimedia, 6136–6145.

[B40] Zhou Y. Barnes C. Lu J. Yang J. Li H. (2019). On the continuity of rotation representations in neural networks, in Proceedings of the IEEE/CVF Conference on Computer Vision and Pattern Recognition, 5745–5753.

